# Karyotypes of Brazilian non-volant small mammals (Didelphidae and Rodentia): An online tool for accessing the chromosomal diversity

**DOI:** 10.1590/1678-4685-GMB-2017-0131

**Published:** 2018-06-28

**Authors:** Roberta Paresque, Jocilene da Silva Rodrigues, Kelli Beltrame Righetti

**Affiliations:** 1 Departamento de Ciências da Saúde, Centro Universitário Norte do Espírito Santo, Universidade Federal do Espírito Santo, São Mateus, ES, Brazil; 2 Departamento de Ciências Agrárias e Biológicas, Centro Universitário Norte do Espírito Santo, Universidade Federal do Espírito Santo, São Mateus, ES, Brazil

**Keywords:** Karyotype diversity, cytogenetic, cytogenetic database

## Abstract

We have created a database system named CIPEMAB (CItogenética dos PEquenos MAmíferos Brasileiros) to assemble images of the chromosomes of Brazilian small mammals (Rodents and Marsupials). It includes karyotype information, such as diploid number, karyotype features, idiograms, and sexual chromosomes characteristics. CIPEMAB facilitates quick sharing of information on chromosome research among cytogeneticists as well as researchers in other fields. The database contains more than 300 microscopic images, including karyotypic images obtained from 182 species of small mammals from the literature. Researchers can browse the contents of the database online (http://www.citogenetica.ufes.br). The system enables users to locate images of interest by taxa, and to display the document with detailed information on species names, authors, year of the species publication, and karyotypes pictures in different colorations. CIPEMAB has a wide range of applications, such as comparing various karyotypes of Brazilian species and identifying manuscripts of interest.

The majority of the karyotypes for Brazilian small mammals are now available. Researchers from multiple fields (e.g., systematicians, ecologists and cytogeneticists) are likely to have interest in some aspects of this data. The web page described here will helps researchers to access Brazilian small mammal karyotype data.

Cytogenetic research addressing Brazilian non-volant small mammals has been continuous since the 1970s. However, karyotype records are dispersed among hundreds of journal articles, often with narrow taxonomic (e.g., Leite *et al.*, 2008; Percequillo *et al.*, 2008), genetic (e.g., Svartman and Viana-Morgante, 1999; Fagundes *et al.*, 2000; Di-Nizo *et al.*, 2015), or geographic focus (e.g., Geise *et al.*, 2010; Di-Nizo *et al.*, 2014). This has made it arduous to analyze large-scale patterns of karyotype evolution across small mammals, or even define which data is available for a clade. To address this, we created the Brazilian small mammal karyotype database (http://www.citogenetica.ufes.br). The database currently contains 182 records, but we envision it as a long-term repository that will be regularly updated. This will allow open access to data that were previously scattered and often available only through subscription based publications.

We stored the karyotype data in a database that can be queried using a dynamically updated webpage. Users can generate database queries by making selections at up to five taxonomic levels (order, family, sub-family, genus or species). Once a user has defined a query, it is used to produce a Portable Document Format (PDF) file. The file allows users to see the karyotype picture in conventional staining or, if available, banding and FISH techniques. It is also possible to download the original article with a karyotype description.

Thus, our web page acts as an atlas of the chromosome complements of Brazilian species, including 147 rodents and 35 marsupials. It is a compilation of all cytogenetic data published considering papers published in scientific journals, abstracts presented at scientific meetings, theses, or other materials of non-restricted access. The web page shows the chromosomal features and options available from the literature, with links to query, display, and download of the data. In addition, this compilation includes the species names, author details, and the year of species publication, as well as karyotype pictures in multiple colorations.

Cytogenetic data for approximately 57% of the rodent species found in Brazil are compiled herein (based on Paglia *et al.*, 2012; Patton *et al.*, 2015). Among the families of the Order Rodentia, we observed a wide variation of diploid and fundamental numbers, wherein the Echimyidae and Cricetidae families had the highest karyotype variation and were also the most specious families in order. Figure 1 shows the magnitude of this variation. In general, for Brazilian rodents, the diploid number ranged from 2n = 10 for *Akodon* sp. from Mato Grosso (Silva and Yonenaga-Yassuda, 1998; Silva *et al.*, 2006) to 2n = 118 for *Dactylomys boliviensis* (Dunnun *et al.*, 2001). Among Sigmodontinae, we observed a pattern showing that the Akodontini tribe has a smaller average 2n compared to Oryzomyini (Figure 1A). A segregation pattern was also observed for terrestrial and semi-fossorial Echimyidae; which showed a 2n smaller than that for arboreal Echimyidae (Figure 1B).

**Figure 1:**
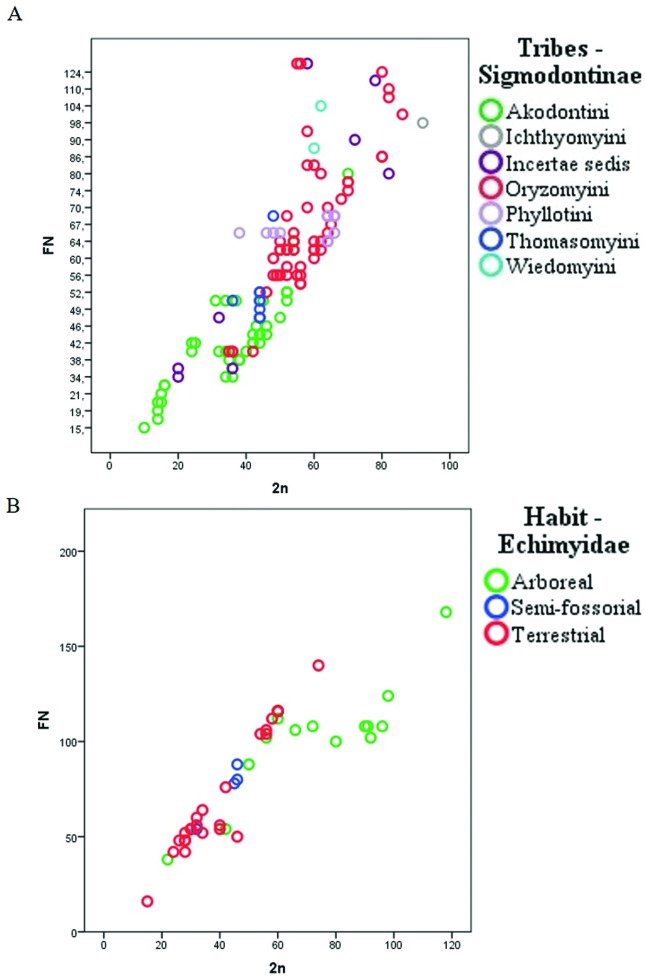
Diploid (2n) and Fundamental numbers (FN) variation between (A) species belonging to the Subfamily Sigmodontinae Wagner, 1843 and (B) species of the family Echimyidae.

The shape of the sex chromosomes also varied. We observed predominant forms of both X and Y chromosomes (the form that appears most often). In Sigmodontinae tribes, the variation in sexual pairs was curious, particularly for the X chromosome (Figure 2). In Oryzomyini and Thomazomyini, in most cases, the X chromosome is large submetacentric (Figure 2A,B), while in Akodontini the predominant form is acrocentric medium (Figure 2C). On the other hand, the Y chromosome is predominantly small acrocentric in Oryzomyini, Thomasomyini, Akodontini, and Phyllotini (Figure 3A-D). In Phyllotini, the X chromosome is highly variable (Figure 2D). Among Echimyidae, the representatives of an arboricolan lifestyle mostly have a large submetacentric X chromosome, whereas those from terrestrial habitats have a medium acrocentric X chromosome (Figure 2E,F). Among Echimyidae, the Y chromosome is small acrocentric, regardless of habitat (Figure 3E,F).

**Figure 2:**
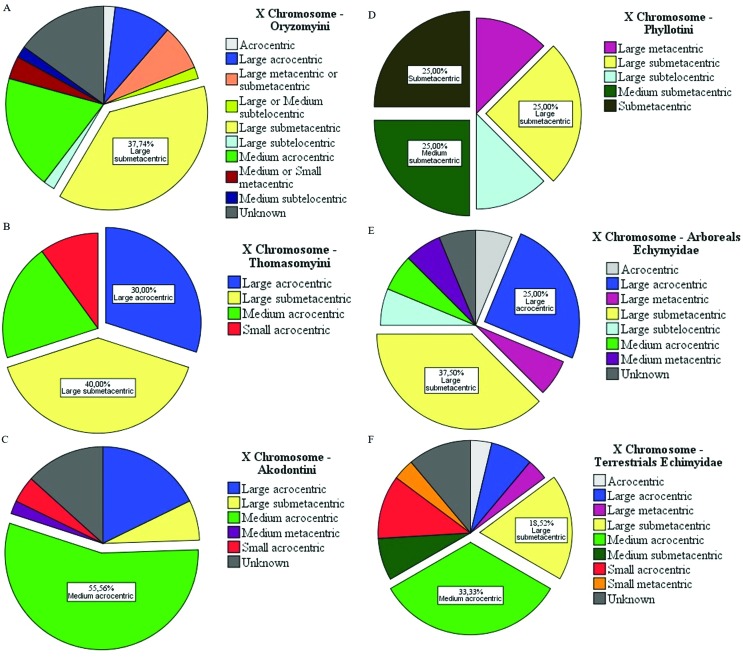
The most usual X chromosomal forms observed for the Sigmodontinae subfamily: (A) Oryzomyini tribe, (B) Thomasomyini tribe, (C) Akodontini tribe, and (D) Phyllotini tribe. The families: Echimyidae (divided by their habits of life) (E) arboreal Echimyidae and (F) terrestrial Echimyidae.

**Figure 3:**
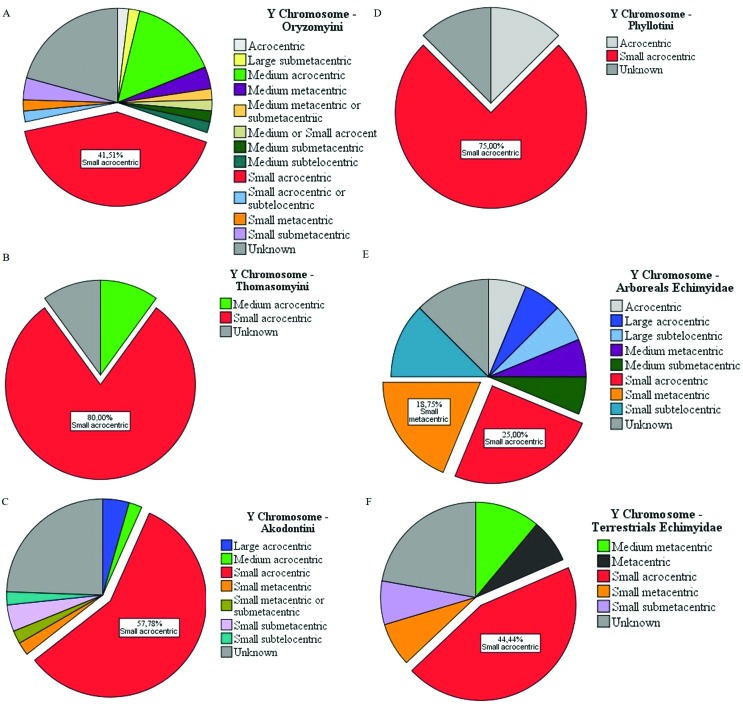
The most usual Y chromosomal forms of the Sigmodontinae subfamily. (A) Oryzomyini tribe, (B) Thomasomyini tribe, (C) Akodontini tribe, and (D) Phyllotini tribe. The families: Echimyidae (divided by their habits of life) (E) arboreal Echimyidae and (F) terrestrial Echimyidae.

We found cytogenetic data for 63% of the 55 marsupial species of the Brazilian territory (according to Paglia *et al.*, 2012). Analyzing the species that show a diploid number of 14 (2n = 14), we observed that the most frequent forms for the X chromosome were small metacentric and small submetacentric (small metacentric: *Marmosa murina, Gracilianus agilis, G. emiliae, Marmosops impavidus, M. incanus, M. noctivagus* and *M. paulensis*; and small submetacentric: *Cryptonanus guahybae, Gracilianus microtarsus, Marmosops neblina, M. parvidens, Thylamys karimii, T. velutinus* and *Caluromys lanatus*) (Figure 4A). The others showed different forms such as small acrocentric in *Metachirus nudicaudatus* and *Cryptonanus agricolai*, and acrocentric or small submetacentric in *Caluromys philander*. The Y chromosome of Brazilian marsupial was mostly small acrocentric (Figure 4D). *Marmosops noctiv*agus has a small metacentric Y chromosome. For *Cryptonanus agricolai, Marmosops neblina*, and *Caluromys philander*, the form of the Y chromosome remains unknown. In relation to the species that showed 2n = 18, the most common form of sexual chromosomes was small acrocentric for both X and Y. Furthermore, they both showed a frequency of 62.50% (Figure 4B). Species that showed a different form of the X chromosome were: small subtelocentric for *Monodelphis scallops,* and small submetacentric for *Monodelphis Kunsi* and *M. dimidiate.* For the Y chromosome, the species that showed different forms were: *Monodelphis domestica, M. brevicaudata* and *M. dimidiate,* which are punctiform (Figure 4E). In those species that are 2n=22, the most common form of the sexual pair are small acrocentric for both X and Y chromosomes (Figure 4C,F). *Lutreolina crassicaudata* has a metacentric form X chromosome. *Chironectes minimus, Didelphis marsupialis,* and *Philander macilhennyi* have unknown Y chromosome forms.

**Figure 4:**
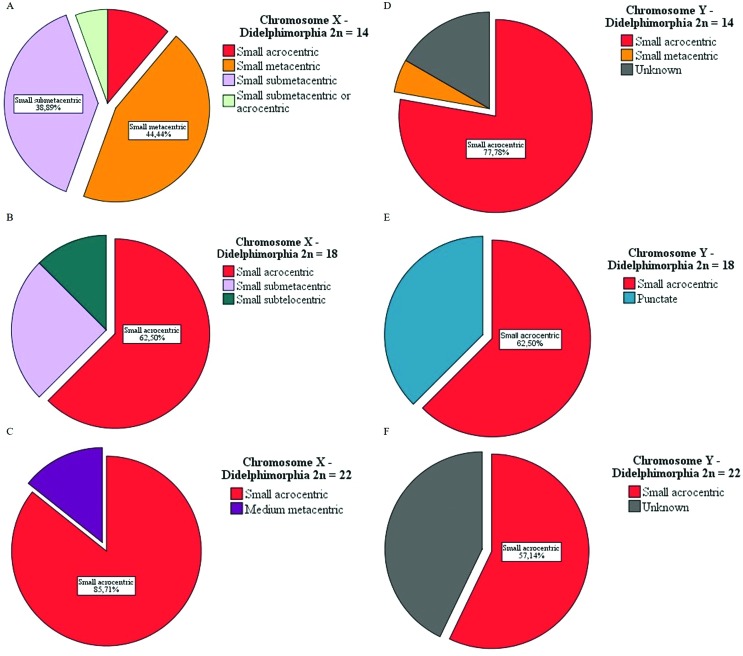
The most common chromosomal forms for the sexual pair found among marsupials. (A and B) 2n = 14 to *Caluromys* sp., *Cryptonanus* sp., *Garcilinanus* sp., *Marmosa* sp., *Marmosops* sp., *Metachirus* sp., *Micoureus* sp. and *Thylamys* sp., (C and D) 2n = 18 to *Glironia* sp. and *Monodelphis* sp., and (E and F) 2n = 22 to *Chironectes* sp., *Didelphis* sp., *Lutreolina* sp. and *Philander* sp.

We show that chromosome number among rodent clades is highly heterogeneous; in most cases the karyotype is species-specific. Therefore, karyotypes remain valuable sources of data. Karyotypes are a highly variable and complex trait that offers an opportunity to detect changes in genome organization, uncover phylogenetic history, and distinguish cryptic species. On the other hand, Brazilian marsupials have just three diploid number variants. However, we detected greater divergence in autosomal number, which in some cases is species-specific.

In recent years, classical comparative cytogenetic and molecular cytogenetic studies have highlighted many fundamental aspects of genome organization and evolution among Brazilian small mammals, providing evidence for the occurrence of fusion, split, and Robertsonian translocations, as well as inversion events involving rodent and marsupial chromosomes. Specific markers for the rodent and marsupial chromosomes were used to explain the evolutionary process associated with the diversification (for Didelphidae examples see Svartman and Vianna-Morgante, 1999; for rodents see Di-Nizo *et al.*, 2015).

By comparing the karyotypes tendencies to Akodotini and Oryzomyini, we proposed two alternative hypotheses to explain the segregated architecture of the 2n and FN these rodents. The first hypothesis assumes that the ancestors of sigmodontines had large chromosomes, similar to those observed in Oryzomyini. Therefore, two independent fragmentation events would have occurred, one in the lineage leading to Akodontini and another leading to Oryzomyini. Alternatively, if the ancestral state corresponded to smaller chromosomes, only one event had to occur, a chromosomal fusion in the lineage leading to Akodontini and Oryzomyini diversifications. Available information cannot determine which of these two hypotheses is most likely. However, the results presented here are in favor of the latter hypothesis. The data available in the literature confirm the occurrence of fusion and split events involving sigmodontine chromosomes and suggest that the common ancestor of rodents had a high chromosomal number. During speciation, these chromosomes joined in different combinations, forming different genomes. It is likely that there is a selective pressure to maintain gene order, although several karyotypic changes could be genetically neutral. Similarly, these events could also explain the karyotype diversity among the rats, where we find a similar segregation pattern. In future studies, data from chromosomal mapping and karyotyping will integrate with genome sequence data. Such integrated maps facilitate draft genome assembly and will be valuable for comparative genomics of rodents.

In summary, the non-volant small mammals most probably represent the mammalian clades, although further studies are needed to confirm this. The studies cited here impact various fields, including comparative genomics, taxonomy, and phylogeography, as well as species conservation and management. This work aimed to provide fundamental insights into the taxonomical principles that may have shaped the specie-specific karyotypes of extant rodents and marsupials, and assist with Rodentia identification. While we will continue to update the database with newly published karyotypes, we also welcome direct contributions and corrections. Relevant data can be submitted to filogenialab@gmail.com.
